# Central Pontine Myelinolysis Induced by Rapid Correction of Hyponatremia in a Patient With Chronic Alcohol Use Disorder: A Case Report

**DOI:** 10.7759/cureus.72521

**Published:** 2024-10-28

**Authors:** Amjad M Mohamadiyeh, Sajad J Allami, Liza Thomas, Uzma Sabahat

**Affiliations:** 1 Medicine, Mohammed Bin Rashid University of Medicine and Health Sciences, Dubai, ARE; 2 Medicine, Kuwaiti Hospital, Sharjah, ARE; 3 Internal Medicine, Dubai Health, Dubai, ARE

**Keywords:** alcoholic withdrawal, central pontine myelinolysis (cpm), chronic alcoholic, cpk, demyelinating disorder, hyponatremia, sodium correction

## Abstract

Central pontine myelinolysis (CPM) is a demyelinating disorder often associated with the rapid correction of hyponatremia. Elevated creatine phosphokinase (CPK) levels have been observed in some cases of CPM but are not well studied. A 38-year-old patient with chronic alcohol use disorder presented with jerky movements, confusion, and disorientation following severe hyponatremia. Initial lab results revealed severe hyponatremia (113 mmol/L) (reference range: 136-145 mmol/L), elevated liver enzymes, and extremely high CPK levels (up to 92,763 U/L) (reference range: <190 U/L). Imaging showed bilateral thalamic and pontine hypodensities, with MRI confirming CPM in the central pons. This case highlights the risk of CPM due to rapid sodium correction in patients with chronic alcohol use disorder and suggests that elevated CPK levels may be associated with CPM. The patient’s condition improved with gradual sodium correction and alcohol withdrawal management. This underscores the importance of careful electrolyte management to prevent CPM and suggests further investigation into the role of highly elevated CPK in CPM. The occurrence of CPM in a patient with chronic alcohol use disorder and elevated CPK levels emphasizes the need for cautious sodium correction, with the association between elevated CPK and CPM warranting further study.

## Introduction

Central pontine myelinolysis (CPM) is a demyelinating disorder characterized primarily by the loss of the myelin sheath in the central basis pons. The main cause of CPM is the rapid correction of hyponatremia [1]. Hyponatremia is the most frequently observed electrolyte disorder in clinical practice, affecting 15%-30% of cases [2]. Rapid correction of hyponatremia may exceed the brain's capacity to restore depleted organic osmolytes, resulting in the development of osmotic demyelination [3,4]. Suspicion of myelinolysis can be confirmed by MRI, which typically demonstrates demyelination in the pons, cerebellum, thalamus, and external capsules [5]. In this case report, we present a case of CPM induced by severe hyponatremia in a patient undergoing alcohol withdrawal. Only a few cases of CPM in alcohol withdrawal patients have been reported in the literature [6].

## Case presentation

A 38-year-old male was admitted to the hospital after experiencing jerky movements in his arms and legs, witnessed by a friend. The patient did not exhibit tongue biting, upward rolling of the eyes, or urinary incontinence. The episode lasted for one minute, followed by 20 minutes of confusion and disorientation. He had no history of chronic illnesses and was not on any medications. However, the patient had been a chronic consumer of beer for the past 20 years, with his last drink occurring three days before the onset of symptoms.

Upon admission, the patient was vitally stable, fully oriented, and conscious. His abdomen was soft and non-tender, with an enlarged liver. Neurological examination was initially unremarkable. Cranial nerves were intact, with normal tone, strength, sensation, and gait. Seventeen days after admission, the patient developed hypertonia and hyperreflexia in all limbs, more prominent on the left side and more severe in the lower limbs than the upper limbs. Motor power was 3/5 in all limbs, with clonus in the right foot. Sensory function and cranial nerves remained intact. The patient also displayed an unsteady, ataxic gait, accompanied by mild tremors. During the first two weeks of admission, he was not mobilizing due to being restrained because of agitation.

Laboratory tests revealed an unremarkable complete blood count (CBC) and mild hypokalemia (3.2 mmol/L; reference range: 3.3-4.8 mmol/L). Severe hyponatremia (113 mmol/L; reference range: 136-145 mmol/L) was managed with normal saline, resulting in a sodium level increase of 10 mmol/L within the first 24 hours, reaching 123 mmol/L. Sodium levels returned to normal within three days. Liver enzymes were elevated, with alanine aminotransferase (ALT) at 172 U/L (reference range: 0-41 U/L) and aspartate aminotransferase (AST) at 319 U/L (reference range: 0-40 U/L). The patient’s total creatine phosphokinase (CPK) level was 9,544 U/L (reference range: <190 U/L) and peaked at 92,763 U/L after three days. He was treated with aggressive intravenous hydration, and his urine output was continuously monitored via catheter.

The patient experienced alcohol withdrawal symptoms, including tremors and fluctuating confusion, which were initially managed with 10 mg of intravenous diazepam every six hours. The dose was escalated to 20 mg every six hours, with additional as-needed doses of diazepam and haloperidol to control increased agitation and confusion.

**Figure 1:**
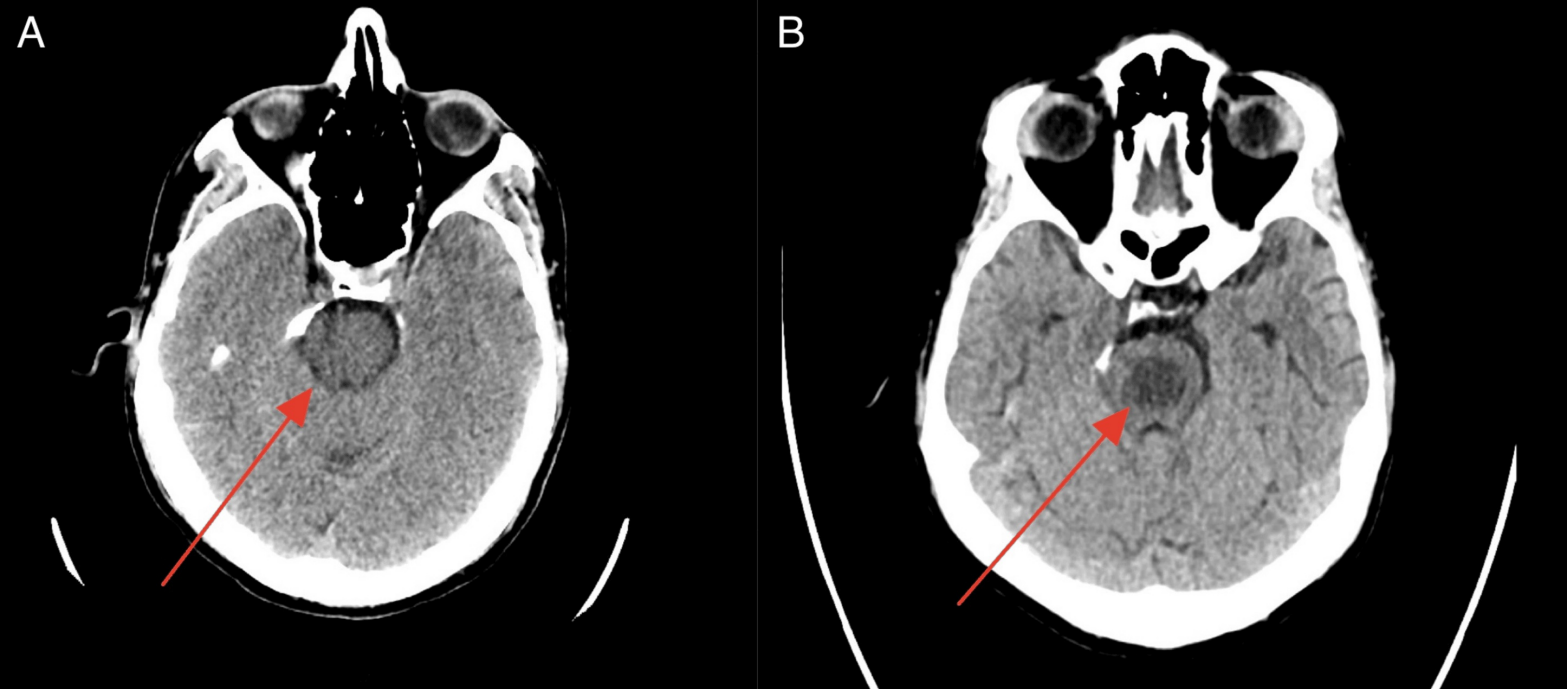
A normal CT scan performed upon the patient’s admission showed no detectable changes in the brain (A), while a follow-up CT scan after sodium correction revealed rounded hypodensities in the pons (B).

A brain CT scan performed upon admission showed no detectable abnormalities. However, after the onset of ataxic symptoms, a repeat CT scan revealed bilateral symmetrical hypodensities in the thalami, rounded hypodensities in the pons, and mild hypodensities in the midbrain and medulla (Figure 1).

**Figure 2:**
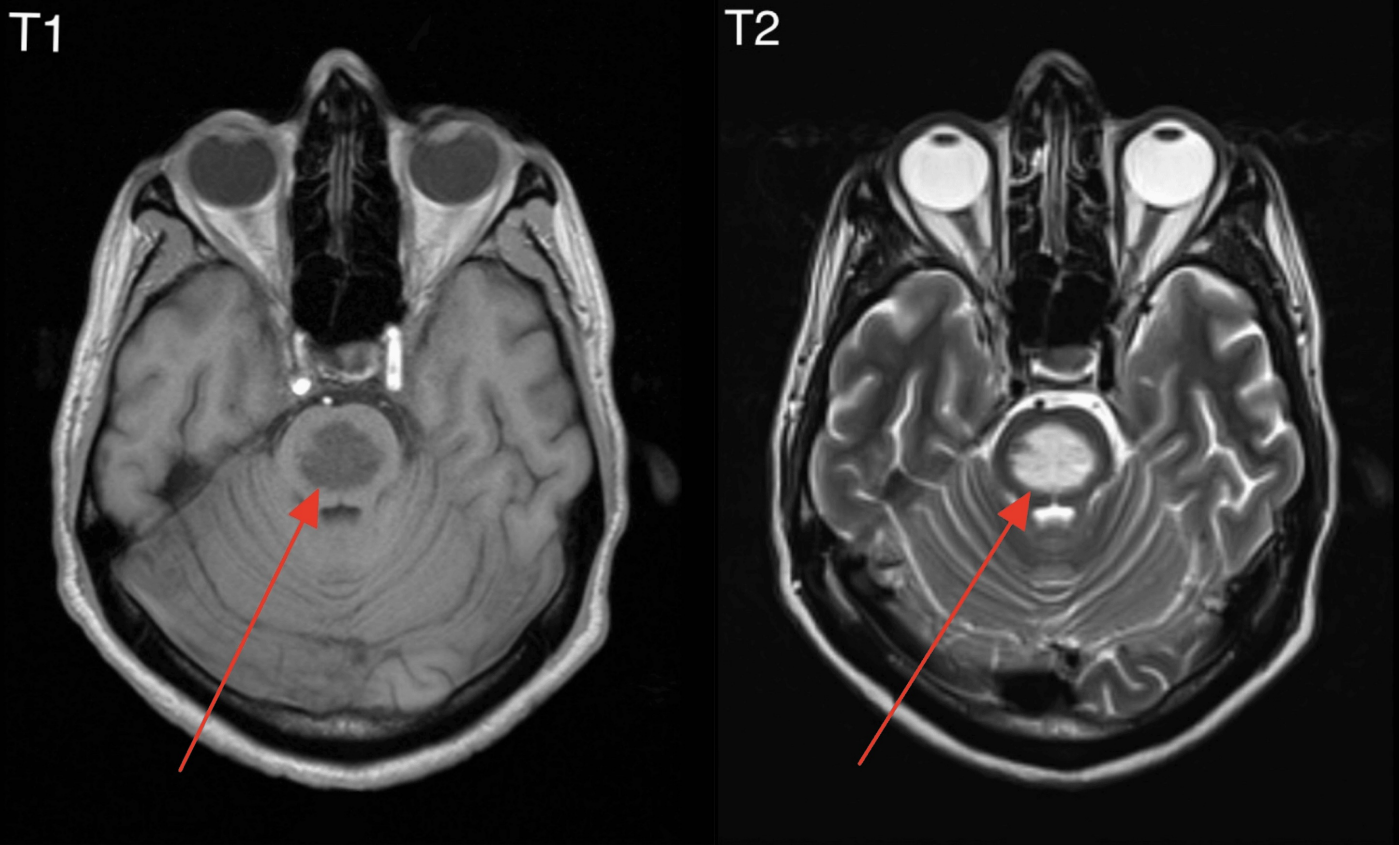
MRI of the brain in T1 and T2 sequences showing a well-demarcated, rounded signal intensity located in the central pons, sparing the peripheries, specifically the ventrolateral longitudinal fibers and corticospinal tracts.

A brain MRI was performed following the onset of ataxic symptoms, along with a CT scan. The MRI revealed a well-demarcated, rounded signal intensity located in the central pons, sparing the peripheries, specifically the ventrolateral longitudinal fibers and corticospinal tracts. This finding was highly suggestive of CPM. No other focal lesions were identified (Figure 2).

In summary, a 38-year-old male with chronic alcohol use disorder presented with jerky movements followed by fluctuating levels of confusion, and later developed hypertonia, hyperreflexia, and ataxia. Laboratory findings revealed severe hyponatremia, elevated liver enzymes, and markedly elevated CPK levels (92,763 U/L; reference range: <190 U/L). Management included aggressive intravenous hydration and diazepam for alcohol withdrawal symptoms. Imaging showed bilateral symmetrical hypodensities in the thalami, rounded hypodensities in the pons, and mild hypodensities in the midbrain and medulla on the brain CT, consistent with CPM. MRI confirmed CPM, demonstrating a well-demarcated lesion in the central pons.

After 24 days of admission, the patient’s functionality improved with the early initiation of daily physiotherapy. His confusion resolved, and he began mobilizing with support, though he still exhibited a mild ataxic gait. The patient was discharged to his home country with a tapering dose of lorazepam over six days, Neurobion twice daily, and was advised to quit alcohol and continue physiotherapy and rehabilitation.

## Discussion

CPM is primarily triggered by the rapid correction of sodium levels. The normal process of correcting hyponatremia involves an increase in extracellular tonicity, followed by a corresponding elevation in intracellular tonicity. During prolonged hyponatremia, the brain compensates by reducing intracellular levels of osmolytes to maintain isotonicity with its surroundings. However, the pathophysiology of CPM arises when sodium levels are corrected too quickly, and the brain fails to adjust to the new tonicity balance between extracellular and intracellular compartments. As a result, osmolytes continue to exit brain cells, leading to the separation of axons from their myelin sheath [6,7].

The primary etiology of CPM in patients with chronic alcohol use disorder is malnutrition, which places these individuals at high risk of deficiencies in essential osmolytes such as sodium, potassium, and phosphates, thereby increasing their susceptibility to demyelination syndromes [8].

Chronic alcohol abuse appears to be a significant risk factor for CPM and extrapontine myelinolysis (EPM). Reports indicate that individuals with chronic alcohol use may present with mild or even no symptoms and generally have better outcomes than cases associated with acute correction of hyponatremia [6]. Supporting this, our patient was able to walk with minimal support despite his ataxic gait and was fit to travel to his home country, with advice for continued physiotherapy and follow-up.

MRI is more sensitive than CT for detecting demyelination lesions [5]. However, both CT and MRI may fail to detect myelinolytic lesions within the first two weeks of symptom onset. Additionally, imaging findings of CPM may resemble those of brainstem ischemic lesions on CT or gliomas on MRI [9], highlighting the importance of correlating clinical presentation with radiological findings.

Clinical outcomes in CPM cases vary, ranging from moderate motor changes to severe locked-in syndrome in cases involving the pontine tegmentum [10]. The appropriate rate of sodium correction to prevent neurological complications has been debated for decades. According to the European Clinical Practice Guidelines, sodium correction should not exceed 10 mEq/L within the first 24 hours and 8 mEq/L per day thereafter. However, several documented cases of osmotic demyelination syndrome (ODS) have adhered to these guidelines, yet complications still occurred [11,12]. Correction of hypokalemia can also lead to an overly rapid correction of hyponatremia, contributing to ODS. In our case, the patient’s mild hypokalemia (3.2 mmol/L; reference range: 3.3-4.8 mmol/L) was corrected to 4 mmol/L, coinciding with the correction of sodium levels.

Although there appears to be an association between CPM and elevated CPK levels in multiple case reports, a comprehensive meta-analysis or systematic review has not yet been conducted to thoroughly investigate this relationship. Understanding this association could provide valuable insights into prognosis.

## Conclusions

In summary, we presented the case of a 38-year-old male with chronic alcohol use disorder who exhibited jerky movements followed by fluctuating levels of confusion and later developed hypertonia, hyperreflexia, and ataxia after the initiation of treatment. Laboratory results revealed severe hyponatremia (113 mmol/L), elevated liver enzymes, and rising CPK levels (from 9,544 U/L to 92,763 U/L). Management included aggressive intravenous fluids and diazepam. Initial imaging was unremarkable; however, following the onset of ataxia, a subsequent brain MRI revealed findings highly suggestive of CPM. Once the patient’s confusion and functionality improved, physiotherapy was initiated. He was able to mobilize with assistance, exhibiting a mild ataxic gait. The patient was discharged on a tapering dose of iorazepam and neurobion and was advised to quit alcohol and continue physiotherapy and rehabilitation.

To conclude, based on findings in the literature and in our case, it may be prudent to adopt a more conservative approach to hyponatremia correction than currently recommended guidelines suggest for high-risk patient populations. As recommended by some experts, we suggest limiting the rate of sodium correction to less than (not equal to) 8 mEq/L within any 24-hour period to minimize the risk of ODS.
